# Crystal Structure of an (*R*)-Selective ω-Transaminase from *Aspergillus terreus*

**DOI:** 10.1371/journal.pone.0087350

**Published:** 2014-01-30

**Authors:** Andrzej Łyskowski, Christian Gruber, Georg Steinkellner, Martin Schürmann, Helmut Schwab, Karl Gruber, Kerstin Steiner

**Affiliations:** 1 ACIB GmbH, c/o TU Graz, Graz, Austria; 2 DSM, Innovative Synthesis, Geleen, The Netherlands; 3 Institute of Molecular Biotechnology, TU Graz, Graz, Austria; 4 Institute of Molecular Biosciences, University of Graz, Graz, Austria; Institute of Enzymology of the Hungarian Academy of Science, Hungary

## Abstract

Chiral amines are important building blocks for the synthesis of pharmaceutical products, fine chemicals, and agrochemicals. ω-Transaminases are able to directly synthesize enantiopure chiral amines by catalysing the transfer of an amino group from a primary amino donor to a carbonyl acceptor with pyridoxal 5′-phosphate (PLP) as cofactor. In nature, (*S*)-selective amine transaminases are more abundant than the (*R*)-selective enzymes, and therefore more information concerning their structures is available. Here, we present the crystal structure of an (*R*)-ω-transaminase from *Aspergillus terreus* determined by X-ray crystallography at a resolution of 1.6 Å. The structure of the protein is a homodimer that displays the typical class IV fold of PLP-dependent aminotransferases. The PLP-cofactor observed in the structure is present in two states (i) covalently bound to the active site lysine (the internal aldimine form) and (ii) as substrate/product adduct (the external aldimine form) and free lysine. Docking studies revealed that (*R*)-transaminases follow a dual binding mode, in which the large binding pocket can harbour the bulky substituent of the amine or ketone substrate and the α-carboxylate of pyruvate or amino acids, and the small binding pocket accommodates the smaller substituent.

## Introduction

Chiral amines are important building blocks for the synthesis of pharmaceutical products, fine chemicals and agrochemicals [1]–[3]. Although there are several biocatalytic methods available for the production of enantiopure chiral amines via kinetic resolution using hydrolases, monoamine oxidases and ω-transaminases [4]–[6], and very recently two amino acid dehydrogenases were engineered to amine dehydrogenases [7], [8], to date ω-transaminases are the only natural enzymes that can directly synthesize enantiopure chiral amines by asymmetric amination of prochiral ketones [9]–[11].

**Figure 1:**
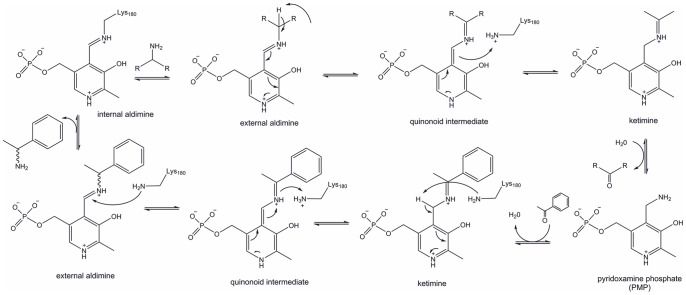
Detailed reaction mechanism of transaminases.

ω-Transaminases catalyse the transfer of an amino group from a primary amino donor to a carbonyl acceptor with pyridoxal 5′-phosphate (PLP) as cofactor. The reaction can be divided into two half reactions, where the amino group is first transferred to PLP to form PMP (pyridoxamine phosphate) and then from PMP onto the carbonyl group (Figure 1) [12]. In more detail, in the resting state the aldehyde group of PLP forms a Schiff base with the side chain amino group of an active site lysine of the protein (internal aldimine). The amino group of the amino donor should be oriented towards this aldimine to enable a transaldimination reaction between the aldimine and the amine donor resulting in the formation of a Schiff base between PLP and the amino donor. This external aldimine is further converted to a ketimine by a 1,3-prototropic shift. The released lysine acts as catalytic base in the hydrolysis of the ketimine to a ketone, which then leaves the active site. The formed PMP then reacts with a newly bound amine acceptor (ketone) and undergoes reductive amination by reversing the reaction described above. Thereby a new amine product is formed and the internal aldimine is regenerated for another reaction cycle.

For a long time mainly (*S*)-selective ω-transaminases were known and extensively investigated [3], [13]–[17]. Thus, (*R*)-amines were mainly prepared by kinetic resolution of racemic amines by (*S*)-transaminases [18]. However, with this method (*R*)-amines are obtained only with a maximum yield of 50%. Recently, several (*R*)-selective enzymes have been described and are under investigation regarding their substrate scope [19]–[22]. Moreover, successful engineering approaches have been described to broaden the substrate scope of (*R*)-transaminases towards bulky substrates such as Sitagliptin [23]. The (*R*)-ω-transaminase from *Aspergillus terreus* (AT-ωTA) has been shown to preferably convert aliphatic substrates (chain length up to at least six carbons) with high yield and high enantioselectivity. However, the yields are significantly lower with aromatic substrates, especially if the ketone is located next to the aromatic ring, e.g. acetophenone, and decreases further when the methyl is exchanged by larger groups [21], [24].

Several structures of amino acid transaminases have been determined to date. Only recently the first structures of (*S*)-selective amine transaminases from *Vibrio fluvialis* JS17 (PDB ID: 3NUI, 4E3Q and 4E3R, [13], [25]), *Paracoccus denitrificans* (PDB ID: 4GRX, [26]), *Pseudomonas putida* (PDB ID: 3A8U, unpublished), *Pseudomonas aeruginosa* (PDB ID: 4BQ0, 4B9B, 4B98, [27]), and *Chromobacterium violaceum* (PDB ID: 4A6R, 4A6T, 4A6U, 4A72, 4AH3, 4BA4, 4BA5, [27], [28]) have been determined or annotated as (*S*)-selective amine transaminases by analysing structures deposited in the protein database (PDB ID: 3HMU, 3FCR, 3GJU, 3I5T, [29]). During the time of the submission of this manuscript a paper describing the crystallization and preliminary X-ray diffraction of the (*R*)-selective amine transaminase from *Aspergillus fumigatus* has been published [30] and its unreleased structure has been deposited in the PDB (PDB ID: 4CHI).

In this paper we describe the structure of an (*R*)-amine aminotransferase (AT-ωTA) from *Aspergillus terreus*.

## Materials and Methods

### Plasmid Construction

The gene (gene ID: 115385557) encoding the ω-(*R*)-transaminase (XP_001209325, Uniprot: **Q0C8G1**, [19]) from *Aspergillus terreus*, which was codon-optimized for *E. coli,* was ordered from GeneArt (Life Technologies, Carlsbad, CA, USA). The gene was recloned into the pET28a(+) expression vector (Novagen/Merck, Darmstadt, Germany) via the restriction sites *Nco*I and *Xho*I to introduce a C-terminal His-Tag. The pET28a-AT-ωTA-CHis construct was confirmed by sequencing (LGC Genomics, Berlin, Germany) and transformed into the expression host *E. coli* BL21-Gold(DE3) (Stratagene, La Jolla, CA, USA). Mutations were introduced by overlap-extension PCR, and the PCR products were ligated into the vector pMS470Δ8 [31], using *Nde*I and *Hin*dIII restriction sites (primer sequences see Table S1 in File S1). The constructs were confirmed by sequencing. *E. coli* TOP10F’ cells (Life Technologies) were used as expression host.

### Expression and Purification

*E. coli* BL21-Gold(DE3) cells harbouring pET28a-AT-ωTA-CHis and *E. coli* TOP10F’ cells containing the pMS470-AT-ωTA wild-type [24] and mutants thereof were grown in LB medium (lysogeny broth) supplemented with kanamycin (50 µg/mL) or ampicillin (100 µg/mL), respectively, at 37°C. Expression of recombinant protein was initiated by addition of 0.1 mM IPTG to OD600 ∼0.8 cultures, and cultivation was continued at 25°C for 20 h. Protein expression and localization was monitored by SDS-PAGE. The cells were harvested, resuspended in cold buffer A (20 mM sodium phosphate buffer, pH 7.4, containing 0.1 mM PLP, 0.5 M NaCl and 10 mM imidazole) and disrupted by sonication (Branson Sonifier S-250; 6 min, 80% duty cycle, 70% output). The cell lysate was centrifuged for 1 h at 50,000 g to remove unbroken cells and insoluble material. The cell free lysate was filtered through a 0.45 µm syringe filter and incubated with Ni Sepharose™ 6 Fast Flow resin (GE Healthcare, Uppsala, Sweden) for 20 min. The Ni Sepharose™ resin was then filled into an empty PD-10 column. Impurities were removed by washing with buffer A containing 30 mM imidazole, followed by elution of the bound protein with 300 mM imidazole in buffer A. Fractions were analysed by SDS-PAGE, pooled and concentrated using Vivaspin 20 Centrifugal Filter Units (10,000 Da molecular-weight cut-off; Sartorius) and desalted on PD-10 desalting columns (GE Healthcare) into 20 mM Tris/HCl, 200 mM NaCl, pH 7. The samples were concentrated and frozen until further use.

### Crystallization

The samples were purified by size-exclusion chromatography (SEC) on a Superdex 200 GL 10/300 column (GE Healthcare) equilibrated with a multi-component buffer system (L-malic acid/MES/Tris pH 6.5, [32]) at 0.1×buffer concentration immediately before crystallization. Peak fractions were pooled, a part of it was set aside and the rest was concentrated to 10 mg/mL using 0.5 mL Amicon Ultra 10K centrifugal filters (Millipore) and used for crystallization trials. Two drops with a total volume of 600 nL (1∶1 ratio of protein solution to screen solution) were dispensed using an Oryx8 protein crystallization robot (Douglas Instruments Ltd) at room temperature (20°C) into a SWISSCi three-well crystallization plate in a sitting-drop vapour-diffusion experiment. The drops consisted of (i) concentrated AT-ωTA sample at 10 mg/mL or (ii) AT-ωTA sample (pooled fractions after SEC) at 3.2 mg/mL and the Morpheus crystallization screen (Molecular Dimensions, [33]). After the initial setup, the crystallization plate was stored at 18°C and visually inspected at time intervals. Crystals began to appear within days and were harvested within a week after the experiment set-up. Two conditions produced crystals of sufficient size for structure determination: B02 with 10% w/v PEG 8000, 20% v/v ethylene glycol, 0.03 M of each halide (sodium fluoride, sodium bromide, sodium iodide) in 0.1 M MES/imidazole, pH 6.5, and H02 with 10% w/v PEG 8000, 20% v/v ethylene glycol, 0.2 M each amino acid (sodium L-glutamate, DL-alanine, glycine, DL-lysine HCl, DL-serine) in 0.1 M MES/imidazole, pH 6.5. Data for structure determination were collected from crystals harvested from condition H02 without cryoprotection.

### Data Collection, Processing and Structure Determination

A data set sufficient for structure determination was collected at beam line PXIII (X06DA) of a third-generation synchrotron light source, the Swiss Light Source (SLS) at the Paul Scherrer Institute. The crystal diffracted to 1.63 Å as determined by the automated data reduction software xia2 used for image processing (3d mode with XDS and XSCALE used for direct image processing and Scala for merging [34], [35]). The initial molecular replacement (MR) solution was obtained with PHASER (version 1.7.2_869, [36]) using a multiple template ensemble. The ensemble was constructed using phenix.ensembler [36] in automated mode from related structures available from the PDB data bank, which were identified by sequence similarity. The initial solution was automatically rebuild with the AutoBuild Wizard from the Phenix suite and further refined with phenix.refine [36], and manual building cycles with Coot [37]. The final refinement round was performed with phenix.refine version 1.8.3–1479. Atomic coordinates and structure factors have been deposited in the Protein Data Bank (PDB ID: 4CE5).

### Docking

Ligand docking calculations were performed using Glide from the Schrödinger 2012 software package (Glide version 5.8, Schrödinger, LLC, New York, [38]) in extra precision mode with extended sampling. The AT-ωTA crystal structure was prepared using the protein preparation wizard by removing bound ligands and alternate amino acid conformations, adding and optimizing hydrogens and modelling of missing side chains. Pro-(*R*) and -(*S*) conformations of the acetophenone-aldimine structure were sketched in Maestro and parameterized for the OPLS/aa force field. The resulting ligand structures were minimized using a steepest descent method before a conformational search was performed yielding the lowest energy conformers that were further used for the subsequent docking protocols. Visualization was done using Maestro (version 9.3, Schrödinger, LLC) and PyMOL (version 1.5.0.5, www.pymol.org).

### Structure Analysis

The refined AT-ωTA structure along with the template structures used for molecular replacement identified using the Swiss-Model Template Identification module (Figure S1 in File S1, [39]) supplemented by a manual selection of other relevant structures were aligned in the program Strap [40] using the Superimpose_CE module (3D structure based alignment). The alignment was then transferred to the program Jalview [41] for further analysis. The phylogenetic tree was generated using the program Archaeopteryx (Ver. 09813, [42]).

In order to identify the conserved regions of the AT-ωTA structure as well as to facilitate the amino acid conservation analysis, the previously described structures were processed using the phenix.ensembler module (ver. 1.8.3–1479, [36]). The templates were separated into single polypeptide chains and aligned according to the module’s standard settings. The conserved portion of the structures was calculated with a standard similarity threshold value of 3.0 Å. In order to reconstruct a functional dimer in a consistent way for all proteins in the alignment, chain A was duplicated and aligned to chain B of AT-ωTA for all analysed structures.

### Activity Assay

The activities of AT-ωTA wild-type and its variants were determined using a standard photometric assay at 25°C [43] containing 0.25 mg/mL of total lysate protein, 0.1 mM PLP, 5 mM *(R)-*α-methylbenzylamine and 5 mM pyruvate or 5 mM butanal in 50 mM KPi, pH 7.5. The increase of acetophenone was measured at 300 nm (extinction coefficient = 0.28 mM^−1^ cm^−1^). As all tested variants expressed at the same expression level as AT-ωTA wild-type, it was feasible to compare the activities of the cleared lysate without purifying the different variants.

## Results and Discussion

Transaminases belong to the fold classes I and IV of (PLP)–dependent enzymes, which are classified into five different protein folds [44], [45]. Fold class I can be further divided into five aminotransferase classes (I-V), which mainly contain L-amino acid aminotransferases. In contrast to α-amino acid aminotransferases (EC 2.6.1), which are ubiquitous enzymes, the small group of amine transaminases also converts substrates lacking an α-carboxylic acid moiety. Within fold class I the (*S*)-selective ω-aminotransferases belong to class III. Fold class IV (PLP)–dependent enzymes comprise D-amino acid aminotransferases (D-ATA, EC 2.6.1.21), branched chain amino acid aminotransferases (BCAT, EC 2.6.1.42) and aminodeoxychorismate lyases (ADCL). Based on homology models [23], [46] of (*R*)-selective ω-aminotransferases, which have been published recently, and due to sequence similarities these enzymes are predicted to belong to fold class IV as well.

### The Overall Structure of AT-ωTA

The crystal structure of AT-ωTA was determined by X-ray crystallography at a resolution of 1.6 Å. Crystallographic data processing and structure refinement statistics are presented in Table 1. The asymmetric unit contains two polypeptide chains which form a stable dimer with a contact surface of 2198 Å^2^ spanning 40 interface residues, which corresponds to 14.6% of the total surface area (PDBSum, [47]). The interface contacts are mostly van der Waals interactions (256 contacts) and hydrogen bonds (18 contacts). All 325 residues are visible in the electron density maps although the three N- and C-terminal residues of both chains were refined with lower occupancy due to their weaker defined electron density. No electron density was observed for the C-terminal His-tag. The electron density in the active centre was well defined where the PLP was expected to bind. Furthermore, extra density was observed at the entrance to the binding site suggesting the presence of a PLP adduct.

**Table 1:** Data collection and refinement statistics.

AT-ωTA	
Wavelength (Å)	0.9793
Resolution range (Å)	41.64–1.63 (1.688–1.63)
Space group	*C*222_1_
Unit cell parameters	a = 105.67
	b = 135.31
	c = 116.41
	α = β = γ = 90.0
Total reflections	770992 (76595)
Unique reflections	103737 (10302)
Multiplicity	7.4 (7.4)
Completeness (%)	99.98 (99.98)
Mean I/sigma(I)	19.47 (2.99)
Wilson B-factor	13.12
R-merge	0.08836 (0.7191)
R-meas	0.09502 (0.0190)
CC1/2	0.999 (0.812)
CC*	1 (0.947)
R-work	0.1386 (0.1921)
R-free	0.1668 (0.2261)
No. of non-hydrogen atoms	6303
macromolecules	5136
ligands	61
water	1106
Protein residues	650
RMS(bonds)	0.007
RMS(angles)	1.09
Ramachandran favored (%)	98
Ramachandran outliers (%)	0.46
Clash score	4.75
Average B-factor	19.4
macromolecules	16.3
ligands	21
solvent	33.7

Values in parentheses are for the highest resolution shell.

Compared to the closest homologous structures identified based on sequence similarity using Swiss-Model Template Identification (PDB ID: 2EIY, a putative branched chain amino acid aminotransferase from *Thermus thermophilus* HB8, sequence identity: 26%, unpublished) and direct 3D-structure comparison using the DALI server (PDB ID: 1IYE, branched-chain amino acid aminotransferase from *E. coli*, [48]) AT-ωTA exhibits the typical aminotransferase class IV fold (InterPro: IPR001544, Pfam: Pf01063) consisting of alpha-beta elements including 2-layer sandwiches and alpha-beta barrels. The above-mentioned structures align with the AT-ωTA structure with a Calpha RMSD of 1.58 Å and 1.26 Å, respectively. The most significant difference between AT-ωTA and its related proteins is the N-terminal alpha-helix spanning the initial 20 amino acids.

A phylogenetic analysis based on structure alignments of selected related proteins suggests a closer relationship of the AT-ωTA to D-ATA represented by the structure of D-amino acid aminotransferase of *Bacillus* sp. (PDB ID: 1A0G) (Figure S2 in File S1).

### Active Site

The electron density in the active site pocket is well defined and interpreted as two distinct states: (i) free Lys180 and Lys180 covalently bound to the pyridoxal-5′-phosphate (PLP) and (ii) N-({3-hydroxy-2-methyl-5-[(phosphonooxy)methyl]pyridin-4-yl}methyl)-D-glutamic acid (ligand code: PGD). Both conformations can be observed in each of the polypeptide chains with minor differences in the occupancy. It has to be noted that the PLP-amino acid adduct could also be interpreted as a combination of any of the amino acids present in the used crystallisation condition (sodium L-glutamate, DL-alanine, glycine, DL-lysine HCl, DL-serine) as the observed density was clearly present, but rather weakly defined. We have selected the glutamic acid adduct, as it best illustrated the length of the observed density without complicating the final structure interpretation.

**Figure 2:**
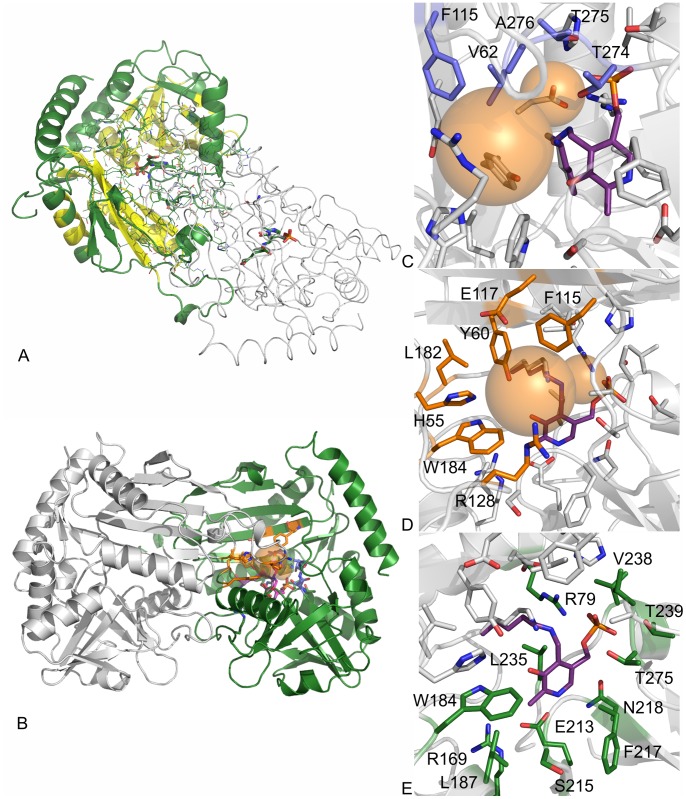
Crystal structure of AT-ωTA. A: Overview of the AT-ωTA dimer (chain A in green, chain B in grey), conserved regions are indicated in yellow, B: overview of the AT-ωTA dimer with the binding pockets indicated as orange spheres, C: small binding pocket amino acids (blue), D: large binding pocket amino acids (orange), E: PLP binding amino acids (green). The figures were prepared using the program PyMOL.

The active site of each polypeptide chain is located on the opposite side of the dimer with the entrance situated at the end of the dimer interface. The entrance cavity is capped by a flexible beta hairpin spanning 15 residues (121–136) thereby limiting the access to the active site. However, none of the hairpin residues belongs to the direct cofactor coordination sphere. There are a total of 125 amino acids within a 12 Å range from the cofactor, 117 belonging to chain A and 8 to chain B. Thereof, 20 amino acids (aa) are located within 0–4 Å. 14 aa are found within a distance of 4–6 Å, 24 aa are 6–8 Å (including His55 from chain B) away, and 67 aa are within a 8–12 Å range, with Gln51, Phe53, Met54, Ser56 and Arg128 (from chain B), as well as Gln183 and Gly185 (from chain A) located along with His55 in the beta hairpin region mentioned above (for a complete list see Table S2 in File S1). Roughly 50% out of those residues are located in the conserved core region of AT-ωTA (Figure 2A, compare Table S2 in File S1). Most of the residues located within a 4 Å range create the lining of the cofactor binding site, with Lys180 directly involved in the activation of the cofactor. Residues located between 6–8 Å are responsible for creating the ‘bottom’ part of the binding pocket while the top layer is created by the residues located between 8–12 Å apart from the cofactor.

Amino acids of both subunits contribute to the formation of the active site of the (*R*)-transaminase from *A. terreus*, which is located at the dimer interface of the two subunits (Figure 2B). These amino acids are building two binding pockets as postulated to represent a general feature of transaminases [9]. The small binding pocket is lined by the amino acids Val62, Phe115, Thr274, Thr275 and Ala276, which interact with the substrate (Figure 2C). The large binding pocket is surrounded by the residues His55*, Tyr60, Phe115, Glu117, Arg128*, Leu182 and Trp184, (*indicates that the residues belongs to the other chain) (Figure 2D); thus, hydrophobic residues and an arginine residue as described for (*S*)-transaminases are present. The arginine residue points towards the carboxylate group of the L-glutamate residue, which is bound to the PMP in the active site.

#### PLP binding site

The electron density for the PLP cofactor is well defined and the covalent imino bond to the active site Lys180 residue is clearly visible.

The phosphate group is interacting through a network of hydrogen bonds to the side chains of Arg79, Thr239 and Thr275, the backbone amides of Val238, Thr239 and Thr275, and two water molecules (Figure 2E). Similar hydrogen networks, which were described as a general feature of the phosphate binding cup of PLP-dependent enzymes [49] have also been observed for other transaminases of both fold classes, in fold class IV, e.g. a D-amino acid transaminase from *Bacillus* sp. YM-1 [50], a branched chain amino acid transaminase from *E. coli* [48], and in fold class I, (*S*)-transaminases e.g. from *P. denitrificans* [26] or *P. aeruginosa* [27], or the aromatic amino acid TA from *P. denitrificans* [51].

An interesting difference between the bindings of the phosphate groups in the two fold classes is the involvement of a highly conserved Arg residue in fold class IV, which is not present in most fold class I TAs.

The pyridine ring of PLP is sandwiched between the side chain of Leu235 and the backbone of Phe217. In transaminases, the electron sink nature of the cofactor is enhanced by a close interaction of the pyridinium nitrogen with a conserved aspartate (fold I) or glutamate residue (fold IV), which helps to maintain the pyridine ring in the protonated form providing resonance stabilization of the carbanionic intermediate [52]. In AT-ωTA, Glu213 takes this role by forming a hydrogen bond between its side chain carboxyl group and the nitrogen atom of the pyridine ring. Glu213 in turn is stabilized by interaction with Arg169 as has also been described for the D-ATA of *Bacillus* sp. [53] and hydrogen bonding of the respective aspartate by a His residues is also observed in fold class I transaminases [27], [49]. The importance of this glutamate residue has also been shown in the D-ATAs by site-directed mutagenesis [54].

### Flexible Loop

**Figure 3:**
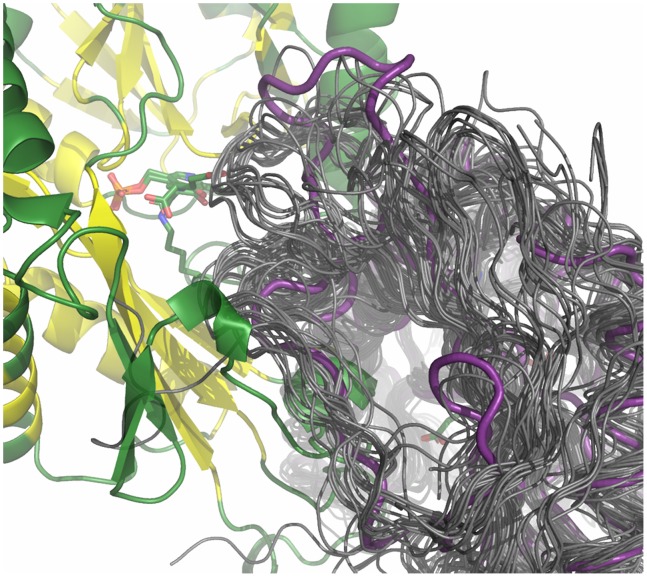
Zoom into the loop Thr121-Val136 region of chain B in the structural alignment of the AT-ωTA structure (magenta) with other fold class IV transaminase structures (dark grey). For PDB-IDs see Figure S2 in File S1. The figure was prepared using the program PyMOL.

As mentioned above, the interdomain loop Thr121-Val136 of chain B is located close to the active site of chain A and vice versa and might function as a flexible lid at the entrance of the active site. Indeed this loop contains several subsequent residues with high B-factors (Arg128-Asp134) indicating a certain degree of flexibility. Interestingly, this loop is not conserved among members of fold class IV transaminases, (Figure S3 in File S1). Moreover, the B-factors of the amino acids in the respective loop of BCATs are much lower. Structural alignments showed that the loops are shorter in many other structures of fold class IV proteins and have different conformations (Figure 3). On the other hand, for the BCAT from *E. coli* a flexible interdomain loop (Pro125-Glu136 corresponding to aa Pro145-Val157 in AT-ωTA), which rearranges upon substrate binding, was reported [48]. The B-factors of this loop are not elevated in AT-ωTA. Structural rearrangements have also been described for the (*S*)-selective transaminase from *Chromobacterium violaceum* [28].

### Docking

**Figure 4:**
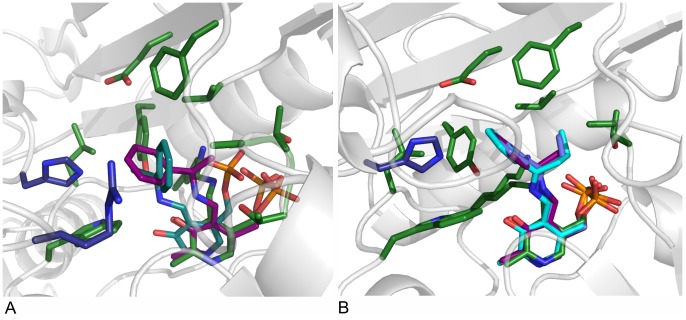
Docking of various substrate intermediates into AT-ωTA. A: Pro-(*R*)- and (*S*)-acetophenone pyridoxal phosphate intermediates docked into the active site of AT-ωTA. Green: amino acids of the active site (chain A) and PLP bound to K180, blue: amino acids of the active site (chain B), purple: pro-(*R*)-acetophenone pyridoxal phosphate intermediate, turquoise: pro-(*S*)-acetophenone pyridoxal phosphate intermediate. B: Acetophenone pyridoxal phosphate intermediate (purple), propiophenone pyridoxal phosphate intermediate (blue), butyrophenone pyridoxal phosphate intermediate (turquoise) docked into the active site of AT-ωTA compared to PLP bound to K180 in the structure of AT-ωTA (green). Green: amino acids of the active site (chain A), blue: amino acids of the active site (chain B). The figures were prepared using the program PyMOL.

Docking of the pro-(*R*)- and (*S*)-ligand conformations of the planar acetophenone pyridoxal phosphate intermediate revealed that only the pro-(*R*) conformer is able to bind to the active site of AT-ωTA in a productive binding mode allowing the attack of Lys180 yielding the (*R*)-enantiomer of the final product (Figure 4A). The hydrophobic aromatic substituent of acetophenone is located in the large binding pocket defined by the mainly hydrophobic residues His55, Tyr60, Phe115 and Trp184, and the methyl group is located in the small binding pocket lined by Val62, Phe115, Thr274, Thr275 and Ala276. To enable the fitting of the acetophenone moiety of the pro-(*S*)-conformation into the large and the small binding pockets without clashing, the PLP moiety gets distorted moving it further away from Lys180. Moreover, especially the phosphate group is displaced (Figure 4A).

Molecular docking of acetophenone-, propiophenone- and butyrophenone-aldimine intermediates confirmed the experimental findings that the size of the small binding pocket influences the binding of larger substrates [21], [24], as butyrophenone can only fit in the active site in a distorted conformation (Figure 4B).

The large binding pocket is open in one direction. This opening leads to a short tunnel, through which the substrates can enter the active site. As it is rather narrow it might influence the entry of larger substrates (Figure S4 in File S1).

### Enantioselectivity

In a recent review, enantiocomplementary enzymes were classified into four different groups based on the position or exchange of active site amino acids in relation to the protein fold or a cofactor as reference [55]. For transaminases two of the groups apply (see below). In group two the protein folds differ, but a cofactor serves as reference point. The positions of the substrates’ substituents are exchanged. Group three comprises enzymes with the same protein fold, but the binding sites of the substrates’ substituents are exchanged as in group two.

#### Binding mode

**Figure 5:**
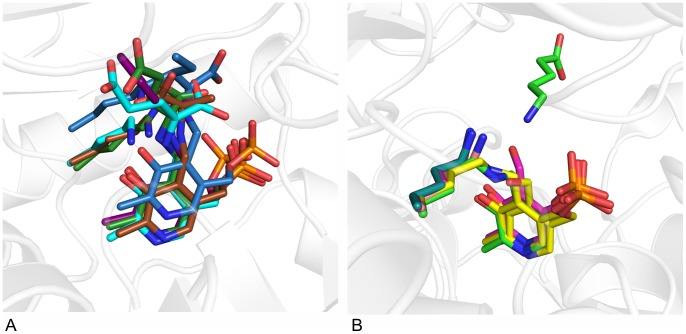
Comparison of fold IV and fold I transaminases. A: Position of lysine relative to PLP in fold IV transaminases: in AT-ωTA (green), BCAT from human (1KT8, blue) or *E. coli* (1IYE, turquoise) and D-ATA from *Bacillus* sp. YM-1 (3DAA, brown). Ligands: blue: L-Ile-aldimine bound in human BCAT, turquoise: L-Glu-aldimine bound in BCAT from *E. coli*, brown: D-Ala-aldimine bound in D-ATA, green: L-Glu-aldimine bound in AT-ωTA, purple: docked acetophenone-aldimine in AT-ωTA. B: Position of lysine relative to PLP in fold I (*S*)-ω-transaminases: in PD-ωTA from *Paracoccus denitrificans* (4GRX, light green), PA-ωTA from *Pseudomonas aeruginosa* (4B98, turquoise) and several (*S*)-TAs identified from the Pdb by Steffen-Munsberg: from *Pseudomonas putida* (3A8U, pink), from *Mesorhizobium loti* (3GJU, yellow) and from *Silicobacter pomeroyi* (3HMU, brownish), in PD-ωTA the substrate, 5-aminopentanoate, is depicted in light green. The figures were prepared using the program PyMOL.

Lysine 180 in AT-ωTA is located at the same position as in the other fold class IV transaminases, BCATs and D-ATAs (e.g. [48], [50]) enabling proton transfer on the *re*-face of PLP (Figure 5A). Moreover, the glutamate residue interacting with the pyridoxal nitrogen and valine/isoleucine, arginine and threonine residues interacting with the phosphate are conserved as well, resulting in an almost identical position of PLP in these three enzymes (Figure S5 in File S1). In contrast, the lysine is positioned on the other side (*si*-face) of the PLP in fold class I proteins like (*S*)-TAs and L-aspartate aminotransferases resulting in a relation of fold I and fold IV enzymes according to group two of enantiocomplementary enzymes (Figure 5B) [55]. The situation that in fold class IV the cofactor is bound in a site that is a mirror image of the fold class I binding sites, so that the *re* rather than the *si*-face is solvent exposed, has already been observed for amino acid transferases almost 20 years ago [53].

In all fold class IV enzymes, the substrates bind at the same face of PLP. Reversed enantioselectivity is achieved by the exchanged localisation of the small and the large binding site and thus the reversed position of the α-carboxylate and side chain of the substrates in D-ATAs and BCATs resulting in either a D-(*R*)-amino acid (D-ATAs), or an L-(*S*)-amino acid (BCAT). Whereas in D-ATAs the small binding pocket accommodating the α-carboxylate is located above the O3’ of PLP, in the BCATs the respective small binding pocket is located above the phosphate group of PLP (Table 2, Figure 5A, compare L-Ile-aldimine bound in BCAT in blue and D-Ala-aldimine bound in D-ATA in brown) [51]. This is in correspondence with group three of enantiocomplementary enzymes [55].

**Table 2:** Localisation of the large and small binding pocket relative to PO_4_ and O3’ of PLP and binding of the substrates’ substituents in different amino acid and amine transaminases (upper part: fold I aminotransferases, lower part: fold IV aminotransferases).

	Large binding pocket^a^			Small binding pocket^a^	
	local	substituent		local	substituent
**L-AroAATA (** ***S*** **) and L-AspTA (** ***S*** **)**	PO_4_	bulky or γ-carboxylate		O3’	α-carboxylate
**(** ***S*** **)-amine TA/L-Ala**	O3’	bulky or α-carboxylate		PO_4_	methyl
**D**-**ATA (** ***R*** **)**	PO_4_	alkyl or γ-carboxylate		O3’	α-carboxylate
**L-BCAT (** ***S*** **)**	O3’	bulky or γ-carboxylate		PO_4_	α-carboxylate
**(** ***R*** **)-amine TA/D-Ala** b	O3’	bulky or α-carboxylate		PO_4_	methyl
**AT-ωTA/L-Glu** c	O3’		γ-carboxylate	PO_4_	α-carboxylate

a defined by the substrate size, in some structures the actual difference in the size of the pockets is very subtle.

b proposed binding as seen in the scheme in Figure 6.

c as observed in the solved structure of AT-ωTA.

For BCATs, a dual substrate recognition for the binding of the γ-carboxylate of glutamate or hydrophobic substrates in the large binding pocket without side-chain rearrangements of active site amino acids has been postulated [19], [48]. The large hydrophobic binding pocket contains hydrophilic sites (e.g. the guanidino group of Arg97, the hydroxy groups of Tyr31* and Tyr129, and the main-chain NH group of Val109* in the BCAT of *E. coli*) close to the solvent region, which interact with the γ-carboxylate of L-glutamate, but do not disturb the binding of hydrophobic substrates e.g. of L-isoleucine, as the inner side of the pocket is mainly hydrophobic. In contrast, in fold class I transaminases, e.g. aspartate (AspTA) and aromatic amino acid transaminases (AroAATA) [56], [57], where the large binding pocket can also harbour either an aromatic or an ω-carboxylate substituent, a different dual binding mode has been described. In the AspTA from *E. coli*, Arg292 in the large binding pocket takes different conformations depending on whether an aromatic or an acidic amino acid binds. It undergoes a movement to a position where it hydrogen-bonds with Asn142, if an aromatic substrate binds [56]. A similar rearrangement is observed in AroTAs [57].

Recently, Bornscheuer’s group used an *in silico* approach to predict key features of a desired (*R*)-ω-transaminase by comparing known crystal structures of BCATs and D-ATAs and identifying conserved amino acids responsible for enantioselective substrate binding, to postulate necessary differences in protein sequences to switch the substrate specificity from α-amino acids to amines [19]. They identified a set of amino acids, which are conserved in a group of highly similar proteins, which indeed displayed the desired (*R*)-selectivity for amines [22].

In accordance with the proposal of Bornscheuer’s group that the plausible ancestor of the (*R*)-specific amine transaminase should be an L-(*S*)-selective BCAT, as, according to the CIP (Cahn-Ingold-Prelog) rule, the substitution of the carboxyl group of an L-amino acid by a methyl group yields an (*R*)-amine, the docking of the acetophenone-aldimine into AT-ωTA placed the methyl group in the small binding pocket and the aromatic ring of the acetophenone in the large binding pocket (Figure 4A). Moreover, as in BCATs the small binding pocket is located above the phosphate group and the large binding pocket above the O3’ (Table 2, Figure 6). Interestingly, L-glutamate is bound in the AT-ωTA structure in exactly the same position as in the BCAT from *E. coli*, 1IYE ([48], Figure 5A), with the γ-carboxylate in the large binding pocket and the α-carboxylate in the small binding pocket. Also several other BCATs with bound substrate show the same geometry (e.g. isoleucine in 1KT8, [58], Figure 5A). However, as expected for an (*R*)-selective enzyme, AT-ωTA is able to use and synthesize several D-amino acids such as D-alanine, D-serine, D-homoalanine, D-threonine as substrates [24], [46], instead of L-amino acids like BCATs. Thus, the methyl group of (*R*)-amines or ketones and the α-carboxylate of amino or keto acids, cannot bind to the same binding pocket, considering the general reaction mechanism for transaminases and taking the priority rule of CIP into account. The α-carboxylate in BCATs is coordinated by the hydroxyl group of a highly conserved tyrosine residue (Y95 in *E. coli* BCAT), which is activated by an adjacent arginine residue (R97 in *E. coli* BCAT, Figure S6A in File S1). These two amino acids are not present in AT-ωTA (F115 and E117, respectively). The other carboxylate oxygen can interact with the backbone amide nitrogen atoms of two residues (e.g. T262 and A263 in *E. coli* BCAT), which are also present in AT-ωTA (T275 and A276). However, an arginine or lysine residue in the second shell of BCATs (R40 in *E. coli* BCAT), which is proposed to activate the backbone amides via coordination with the adjacent carbonyl groups, is missing in AT-ωTA (S64). In summary, the small binding pocket of AT-ωTA and BCAT indeed differ significantly as it was predicted by the group of Bornscheuer to enable the binding of a methyl group instead of the α-carboxylate [19]. In accordance with the complementary enantioselectivity of D-ATAs, the α-carboxylate is bound in the binding pocket above the O3’ of PLP interacting with conserved arginine, histidine and tyrosine residues (Y31, R98, and His100 in the D-ATA from *Bacillus* sp., Figure S6B in File S1). A tyrosine residue is found at exactly the same position in AT-ωTA (Y60) and with His55 and Arg128 positive charged amino acids are present as well (for further analysis see below).

**Figure 6:**
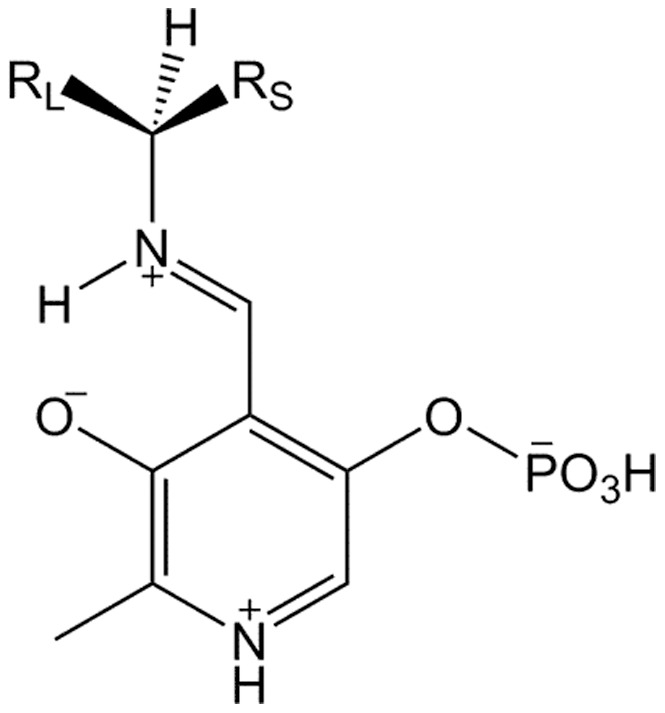
Schematic drawing of the localisation of the large and small binding pocket in AT-ωTA relative to PO_4_ and O3’ of PLP and the binding of the substrates’ substituents.

The binding of L-glutamate in the active site of AT-ωTA, with its α-carboxylate located in the small binding pocket and the γ-carboxylate in the large binding pocket, might be an artefact of the crystallization conditions (see above), which contain a mixture of DL-amino acids. L-Glu does not serve as substrate for AT-ωTA; it would not be converted to the respective keto acid and remained in the active site, whereas D-amino acids might have been converted and thus could not be detected.

A different mode of binding than for BCATs must apply to explain the (*R*)-amine and D-amino acid selectivity of AT-ωTA.

For (*S*)-omega-transaminases a different dual recognition mode than for amino acid aminotransferases has been postulated, in which the bulky substituent of the amine or ketone substrate and the α-carboxylate of pyruvate or amino acids bind in the same (large) binding pocket. In addition, there is a small binding pocket, which prohibits the binding of substituents larger than an ethyl group [16]. With detailed kinetic analysis and calculations of free energies the same group could show that in the (*S*)-transaminase from *P. denitrificans* differences in kcat/Km values of different substrates arise mainly from their different binding affinity than from the catalytic turnover rate [59]. This binding mode was recently also confirmed by analysing solved structures [26], [29]. Docking of α-methylbenzylamine into the structure of an ω-amino acid:pyruvate aminotransferase (PDB ID: 3A8U) from *Pseudomonas putida* indicated that several aromatic amino acids (Tyr 23, Phe88*, Tyr152) in the large binding pocket establish a hydrophobic environment enabling the binding of hydrophobic substituents. In addition, an arginine, R414, is present, which is proposed to interact with carboxylate groups of keto acid substrates [60]. These four residues of the large binding pocket are conserved in other (*S*)-transaminase structures from *P. denitrificans*, *V. fluvialis* and *C. violaceum* as well as in four recently annotated structures (3HMU, 3IST, 3FCR and 3GJU, [29]). Interestingly, Arg417 in 3HMU adopts two different conformations in the two monomers. In subunit A it is oriented away from the PLP and enlarges the binding pocket [60]. Molecular modelling of carboxylic substrates and aromatic substrates revealed that the flexibility of this arginine residue is most likely responsible for the substrate promiscuity. In the crystal structure of *P. denitrificans* the Arg415 residues is found in two orientations dependent on the presence (Arg points outwards) or absence (Arg points in the active site) of the substrate [26].

In AT-ωTA, Arg128 is part of the flexible loop with high B-factors as outlined above. Interestingly, the side chain is slightly kinked out of the active site to create room for the binding of the L-glutamate or alternatively acetophenone, as shown in the docking. Thus, it is probable that Arg128 can move closer to the active site and facilitate the binding of the α-carbonyl of pyruvate in the active site either by changing its conformation or by movement of the flexible loop (Figure S7 in File S1).

**Figure 7:**
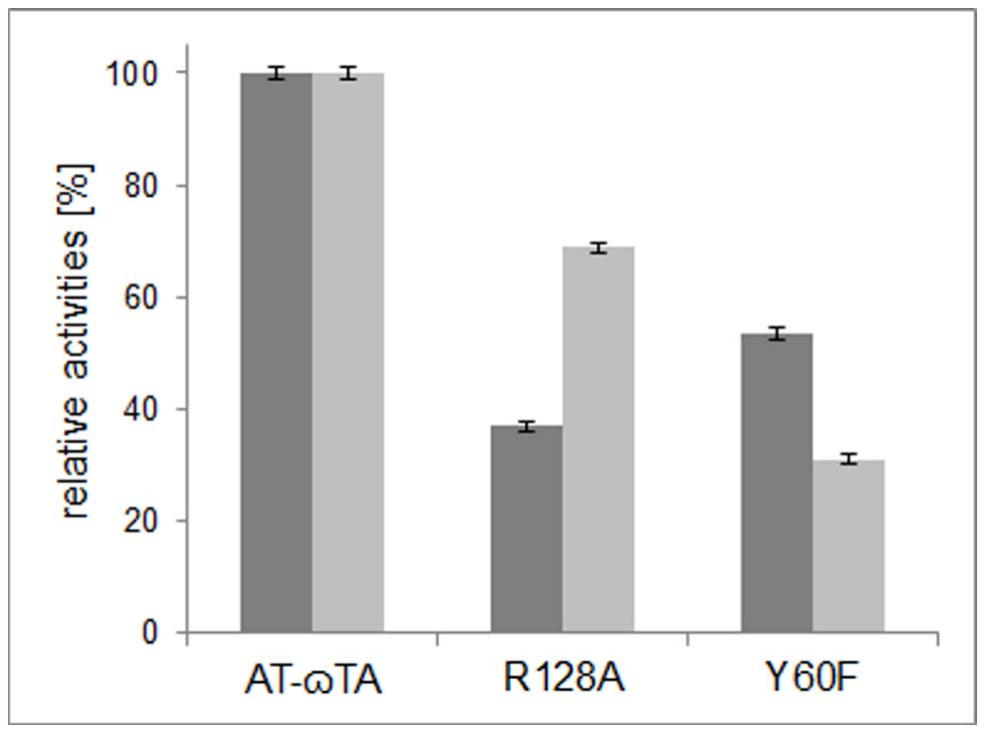
Relative activities calculated from the increase of acetophenone at 300-ωTA and mutants thereof (0.1 mM PLP, 5 mM *(R)-*α-methylbenzylamine, 5 mM pyruvate or 5 mM butanal, in 50 mM KPi, pH 7.5, 0.25 mg/mL of total lysate protein) at 25°C. The relative activities are referred to either the wild-type activity with pyruvate (0.8 U/mg lysate, dark grey bars) or butanal (0.1 U/mg lysate, light grey bars).

The AT-ωTA mutant R128A showed only reduced activity with pyruvate and (*R*)-α-methylbenzylamine (∼37% of wild-type activity (0.8 U/mg), Figure 7) and with butanal the activity was reduced as well (67% of wild-type activity (0.1 U/mg) Figure 7). This is in contrast to the observation in several (*S*)-TAs, where mutations of the above-mentioned arginines resulted in significantly decreased activity towards pyruvate, whereas butanal served still as substrate [61]. The remaining activity of these Arg mutants in (*S*)-TAs was explained with the additional interaction of the carboxylate group with a Trp and Phe residue. In AT-ωTA, Tyr60 and Trp184 may play this role. The mutation of these residues resulted in diminished (Y60A) or completely abolished activity (Y60A, W184A and W184F) with both substrates, pyruvate and butanal (data not shown). Thus, it can well be the case that the hydrophobic aromatic residues in fact interact with the aromatic ring of the methylbenzylamine and the mutations result in inefficient binding of methylbenzylamine.

### Conclusion

After a detailed analysis of the structure of the (*R*)-selective ω-transaminase from *Aspergillus terreus* including a comparison to other members of the fold class IV family and considering the dual binding mode reported for (*S*)-transaminases [16], [61], we propose, that (*R*)-transaminases follow a similar dual binding mode, in which the large binding pocket can harbour the bulky substituent of the amine or ketone substrate and the α-carboxylate of pyruvate or amino acids, and the small binding pocket accommodates the smaller substituent. Co-crystallization experiments with various substrates are currently on-going to obtain a deeper insight into the substrate binding mode.

## Supporting Information

File S1 **Table S1,** Sequences of primers. **Table S2,** (Conserved) amino acids within a 12 Å range from the cofactor PLP in AT-ωTA. **Figure S1,** Output of the Swiss-Model Template Identification module. **Figure S2,** Cladogram of selected fold class IV aminotransferases. **Figure S3,** Sequence alignment of loop Thr121-Val136 of AT-ωTA. **Figure S4,** Amino acids of the entrance tunnel of AT-ωTA. **Figure S5,** Alignment of PLP and PLP-coordinating amino acids of AT-ωTA with other members of the fold class IV family. **Figure S6,** Alignment of active site amino acids of AT-ωTA with other members of the fold class IV family. **Figure S7,** Different rotamers of arginine 128 in AT-ωTA.
(PDF)
