# Supplementary material for: Crystal Structure of an (R)-Selective ω-Transaminase from Aspergillus terreus
Source: PLoS One. 2014 Jan 30;9(1):e87350. doi: 10.1371/journal.pone.0087350 (PMC3907554; doi:10.1371/journal.pone.0087350)
Supplement: File S1 — Table S1, Sequences of primers. Table S2, (Conserved) amino acids within a 12 Å range from the cofactor PLP in AT-ωTA. Figure S1, Output of the Swiss-Model Template Identification module. Figure S2, Cladogram of selected fold class IV aminotransferases. Figure S3, Sequence alignment of loop Thr121-Val136 of AT-ωTA. Figure S4, Amino acids of the entrance tunnel of AT-ωTA. Figure S5, Alignment of PLP and PLP-coordinating amino acids of AT-ωTA with other members of the fold class IV family. Figure S6, Alignment of active site amino acids of AT-ωTA with other members of the fold class IV family. Figure S7, Different rotamers of arginine 128 in AT-ωTA. (PDF) [file pone.0087350.s001.pdf]

SUPPLEMENTAL INFORMATION

Crystal structure of an (*R*)-selective ω-transaminase from *Aspergillus terreus*

Andrzej Łyskowki[1*], Christian Gruber[1], Georg Steinkellner[1], Martin Schürmann[2], Helmut Schwab[1,3], Karl Gruber[1,4], Kerstin Steiner[1*]

Table S1. Sequences of primers used for cloning and mutagenesis in this study. Sequences of restriction sites are underlined, start and stop codon are indicated with boxes and mutated bases are highlighted in bold letters.

Primer name (mutation)	Sequence
AT-ωTA_for(*Nco*I)	AATCACCATGGCGTCTATGGACAAAGTATTCGC
AT-ωTA_rev(*Xho*I-Stop)	AATCACTCGAGATTACGTTCGTTGTAGTCGATTTCGAAGG
AT-ωTA_for(*Nde*I)	AATCACATATGGCGTCTATGGACAAAGTATTCGC
AT-ωTA_rev(*Hind*III)	AATCAAAGCTTATTAATTACGTTCGTTGTAGTCGATTTC
AT-ωTA-Y60A_for	GCACTCTGACCTGACT**GCA**GACGTTCCGTCTGTATG
AT-ωTA-Y60A_rev	CATACAGACGGAACGTC**TGC**AGTCAGGTCAGAGTGC
AT-ωTA-Y60F_for	CTCTGACCTGACTT**T**CGACGTTCCGTCTG
AT-ωTA-Y60F_rev	CAGACGGAACGTCG**A**AAGTCAGGTCAGAG
AT-ωTA-R128A_for	GTCTGAAAGGCGTT**GC**TGGTACTCGTCCG
AT-ωTA-R128A_rev	CGGACGAGTACCA**GC**AACGCCTTTCAGAC
AT-ωTA-W184A_for	GTTAAAAACCTGCAG**GC**GGGCGACCTGGTTC
AT-ωTA-W184A_rev	GAACCAGGTCGCCC**GC**CTGCAGGTTTTTAAC
AT-ωTA-W184F_for	GTTAAAAACCTGCAGT**TC**GGCGACCTGGTTCGTG
AT-ωTA-W184F_rev	CACGAACCAGGTCGCC**GA**ACTGCAGGTTTTTAAC

Table S2. (Conserved) amino acids within a 12 Å range from the cofactor PLP in AT-ωTA.

distance [Å]	chain	residue	conserved
4	A	THR-59	*
4	A	TYR-60	*
4	A	ARG-79	*
4	A	SER-83	*
4	A	THR-178	
4	A	VAL-179	
4	A	LYS-180	
4	A	ASN-181	
4	A	TRP-184	
4	A	GLU-213	*
4	A	GLY-214	*
4	A	GLY-216	*
4	A	PHE-217	*
4	A	ASN-218	*
4	A	LEU-235	
4	A	GLY-237	
4	A	VAL-238	*
4	A	THR-239	*
4	A	THR-274	*
4	A	THR-275	*
6	A	LEU-58	*
6	A	ASP-61	*
6	A	VAL-62	*
6	A	HIS-76	*
6	A	LEU-87	*
6	A	ARG-169	
6	A	ASP-176	
6	A	PRO-177	
6	A	LEU-182	
6	A	LEU-187	
6	A	SER-215	*
6	A	GLN-236	
6	A	ARG-240	*
6	A	CYS-273	*
6	A	ALA-276	*
8	A	PRO-63	*
8	A	SER-64	*
8	A	LEU-80	*
8	A	ALA-82	*
8	A	CYS-84	*
8	A	LYS-86	*
8	A	PHE-115	*

8	A	GLU-117	*
8	A	LEU-118	*
8	A	VAL-120	*
8	A	VAL-149	
8	A	ILE-175	
8	A	GLN-183	
8	A	ASP-186	
8	A	THR-212	*
8	A	ILE-219	*
8	A	GLY-233	
8	A	VAL-234	
8	A	LYS-241	*
8	A	SER-242	*
8	A	GLY-277	
8	A	GLY-278	*
8	A	MET-280	*
8	A	TYR-306	
8	B	HIS-55	
12	A	GLY-52	
12	A	HIS-55	
12	A	SER-56	*
12	A	ASP-57	
12	A	VAL-65	*
12	A	PHE-71	*
12	A	ARG-72	*
12	A	LEU-73	*
12	A	ASP-74	*
12	A	ASP-75	*
12	A	ILE-77	*
12	A	THR-78	*
12	A	GLU-81	*
12	A	THR-85	*
12	A	ARG-88	*
12	A	LEU-89	
12	A	ALA-114	*
12	A	VAL-116	*
12	A	ILE-119	*
12	A	THR-121	*
12	A	ARG-122	*
12	A	ASN-138	*
12	A	TYR-140	*
12	A	PHE-142	*
12	A	GLN-144	*
12	A	TYR-146	

12	A	VAL-147	
12	A	TRP-148	
12	A	MET-150	
12	A	ARG-168	
12	A	VAL-170	
12	A	PRO-171	
12	A	PRO-172	
12	A	ALA-174	
12	A	GLY-185	
12	A	VAL-188	
12	A	ARG-189	
12	A	GLY-190	
12	A	MET-191	
12	A	TYR-201	*
12	A	PRO-202	
12	A	PHE-203	*
12	A	LEU-204	
12	A	THR-205	*
12	A	LEU-211	*
12	A	VAL-220	*
12	A	THR-229	*
12	A	PRO-230	*
12	A	ASP-231	*
12	A	ARG-232	
12	A	VAL-243	*
12	A	ILE-244	*
12	A	ASN-245	*
12	A	PHE-271	*
12	A	MET-272	*
12	A	ILE-279	*
12	A	ILE-302	
12	A	TRP-307	
12	A	MET-309	
12	A	HIS-310	
12	B	GLN-51	
12	B	PHE-53	
12	B	MET-54	
12	B	SER-56	*
12	B	ARG-128	
12	B	GLN-183	
12	B	GLY-185	
12	B	ARG-189	
>12	A	-	ASN-218

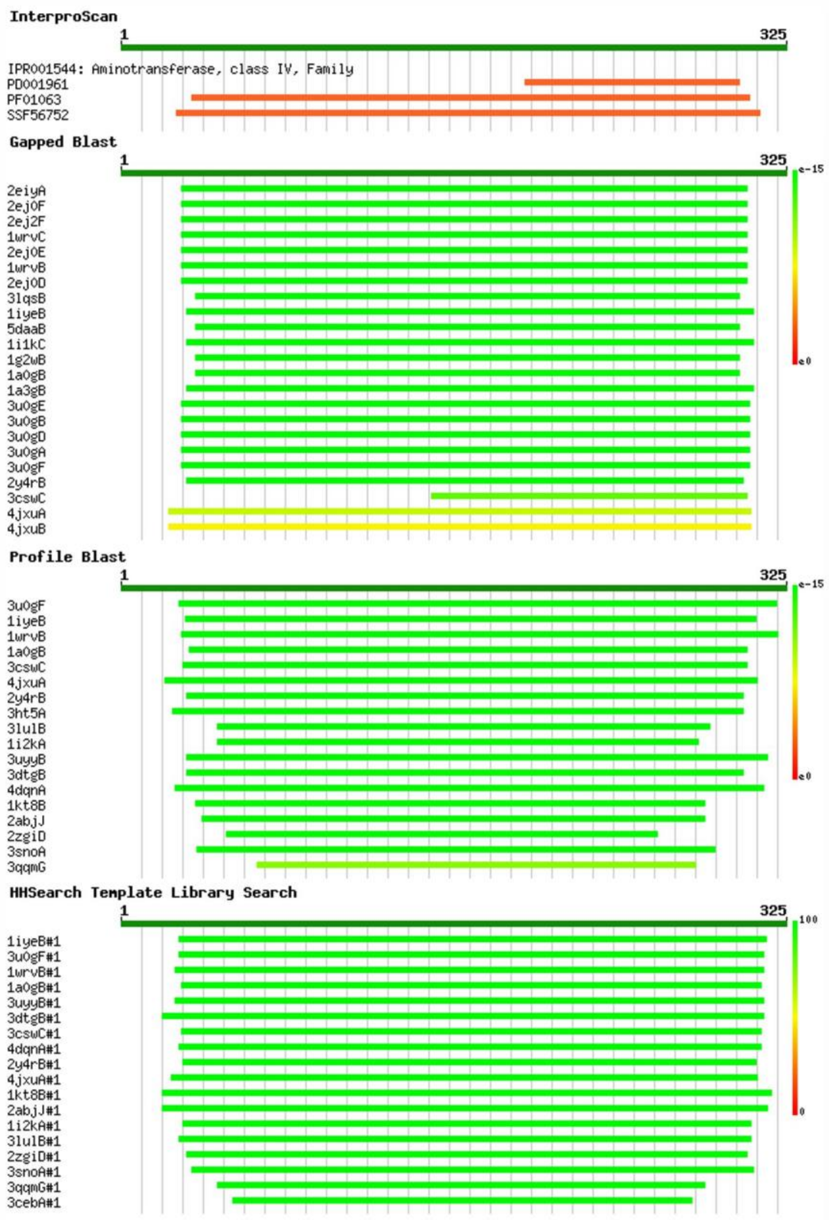

Figure S1. **Output of the Swiss-Model Template Identification module**

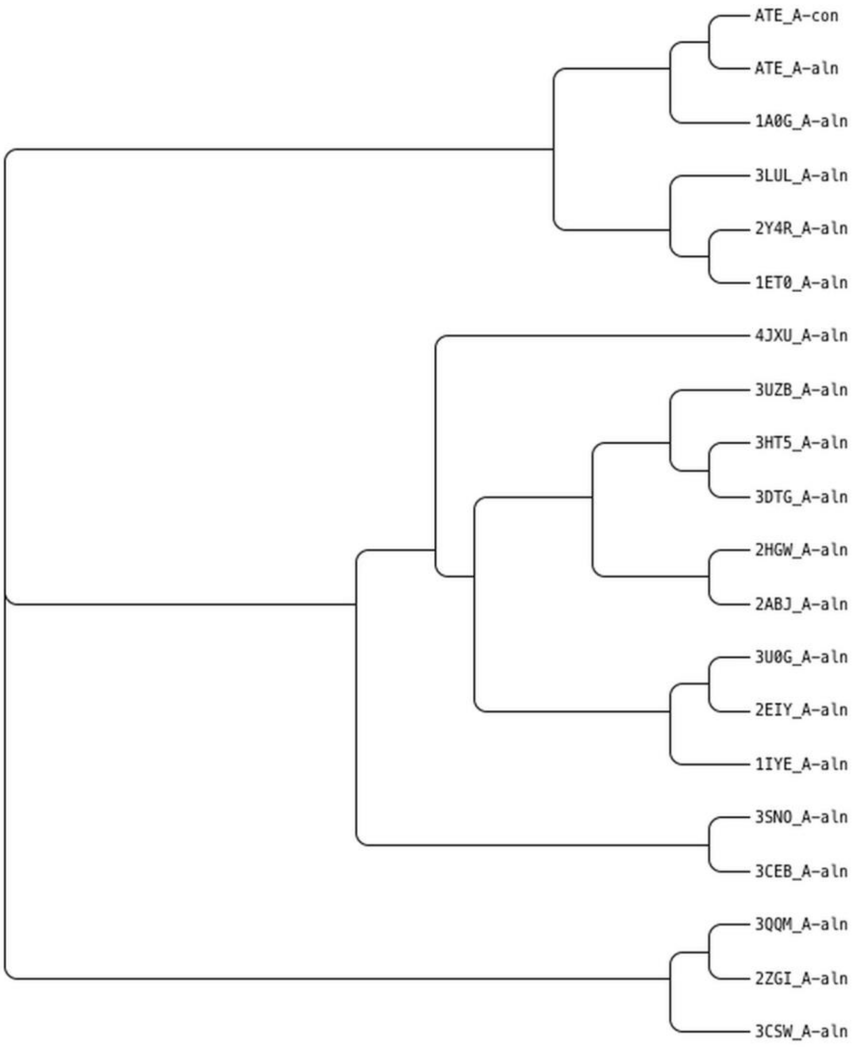

Figure S2. Cladogram of selected fold IV aminotransferases: AT-ωTA, D-ATA: 1A0G from *Bacillus* sp YM-1, BCATs: 1IYE from *E. coli*, 2EIY from *Thermus thermophilus*, 3HT5 from *Mycobacterium tuberculosis*, 3DTG from *Mycobacterium smegmatis*, 3UZB from *Deionococcus radiodurans*, 3U0G from *Burkholderia pseudomallei*, 2ABJ and 2HGW from human, ADCLs: 1ETO from *E. coli*, 2Y4R from *Pseudomonas aeruginosa*, and a few putative aminotransferases: 2ZGI from *Thermus thermophilus* HB8, 3CEB from *Haemophilus somnus*, 3CSW from *Thermotoga maritima*, 3LUL from *Legionella pneumophila*, 3QQM from *Mesorhizobium loti*, 3SNO from *Corynebacterium glutamicum*, 4JXU from *Sinorhizobium meliloti.*

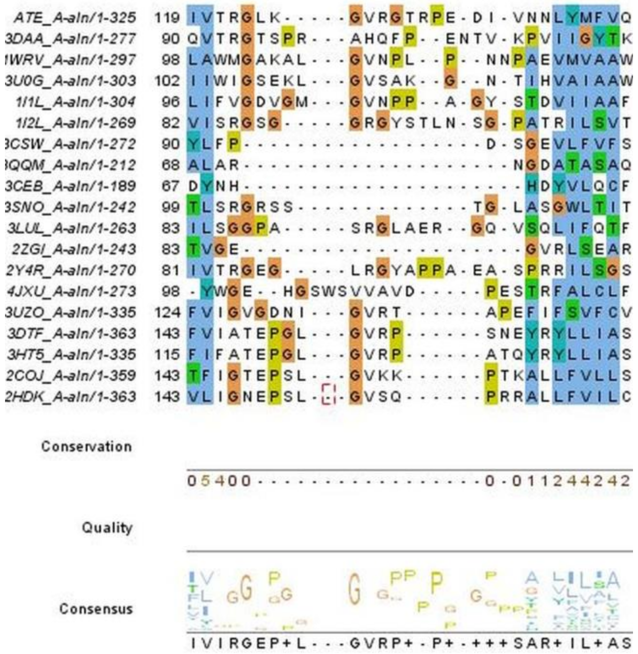

Figure S3. **Sequence alignment of loop Thr121-Val136 of AT-ωTA** to the respective sequences of other fold IV transaminsase structures.

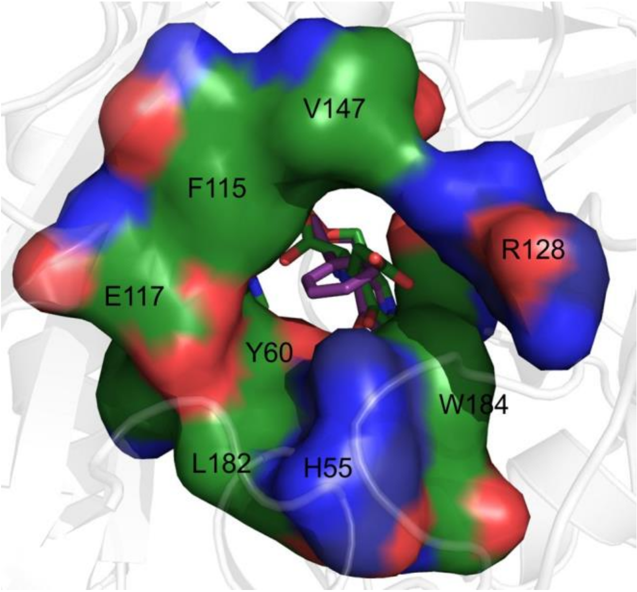

Figure S4. Amino acids of the entrance tunnel of AT-ωTA. Green: chain A, blue: chain B. purple: acetophenone, positioned in the structure by docking, green: L-glutamate, which is bound in the structure. The figure was prepared using the program PyMOL.

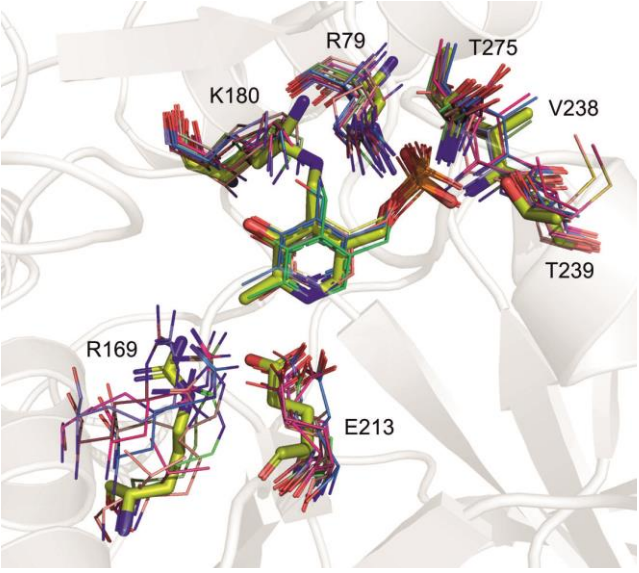

Figure S5. Alignment of PLP and PLP-coordinating amino acids of AT-ωTA (light green sticks) with other members of fold IV family (lines of various colours). The figure was prepared using the program PyMOL.

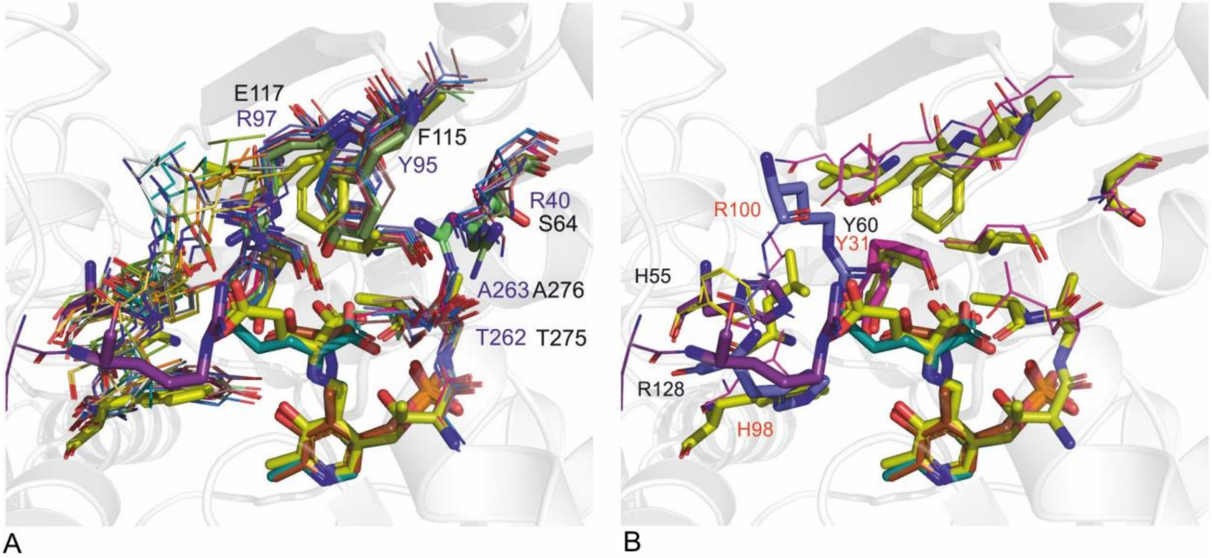

Figure S6. Alignment of active site amino acids of AT-ωTA with other members of the fold IV family (A: BCATs, B: D-ATA). Light green sticks: amino acids and L-Glu-aldimine of AT-ωTA, turquoise: L-Glu-aldimine bound in BCAT, brown: D-Ala-aldimine bound in D-ATA. Other amino acids depicted as sticks: conserved amino acids of either BCAT or D-ATA involved in α-carboxylate binding. Labelling: black: AT-ωTA, blue: BCAT, red: D-ATA. The figures were prepared using the program PyMOL.

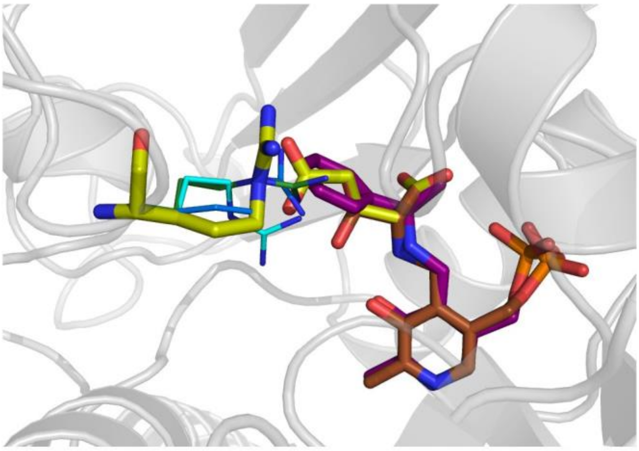

Figure S7. Different rotamers of arginine 128 in AT-ωTA. Grey: Cartoon depiction of the AT-ωTA backbone, light green stick depiction: original conformation of R128 in AT-ωTA; blue, turquoise and green lines: alternative rotamers that move R128 closer to the α-carbonyl of a docked D-Ala-aldimine (brown) and docked acetophenone-aldimine (purple); L-glutamate-aldimine also depicted in light green. The figure was prepared using the program PyMOL.
